# Allergic diseases of the skin and drug allergies – 2026. Immunologic evaluation of the patients with cefaclor hypersensitivity

**DOI:** 10.1186/1939-4551-6-S1-P112

**Published:** 2013-04-23

**Authors:** Hye-Soo Yoo, Seung-Hyun Kim, Hyouk-Soo Kwon, Tae-Bum Kim, Young-Hee Nam, Mi-Ae Kim, Young Min Ye, Hae-Sim Park

**Affiliations:** 1 Department of Allergy & Clinical Immunology, Ajou University School of Medicine, Suwon, South Korea; 2 Division of Allergy and Clinical Immunology, Asan Medical Center, University of Ulsan College of Medicine, Seoul, South Korea; 3 Department of Internal Medicine, College of Medicine, Dong-A University, Busan, South Korea

## Background

Cefaclor is the most common cephalosporin to induce anaphylaxis. We evaluated immunologic findings of cefaclor hypersensitivity.

## Methods

36 patients with a history of immediate reactions to cefaclor were enrolled from Ajou University Hospital and Asan Medical Center, South Korea. Those with immediate hypersensitivity reactions to cefaclor were defined by a certain clinical history with or without specific IgE to cefaclor by immunoCAP system. Serum specific IgE, IgG1 and IgG4 levels to cefaclor-HSA conjugate were measured by ELISA, and compared with those of ImmunoCAP system. The binding specificity was evaluated by ELISA inhibition test.

## Results

Anaphylaxis (group I, 80.6%) was the most common phenotype, followed by urticaria (group II, 19.4%). There were no significant differences in clinical characteristics, such as age, sex and atopy status between group I and II. The serum specific IgE to cefaclor by ImmunoCAP was found in total 29 (80.6%) patients, 24 (82.8%) in group I and 5 (71.4%) in group II with no significant according to clinical parameters. The prevalence of serum specific IgE, and IgG1, IgG4 to cefaclor-HSA conjugate by ELISA tended to be higher in group I (51.7%, 53.6%, 20.7%) than in group II (14.3%, 14.3%, 0%), although these differences were not statistically significant. Serum specific IgG4 to cefaclor-HSA conjugate was observed only in group I. 10.3% patients in group I had high specific IgG1 to cefaclor-HSA conjugate with no specific IgE. Significant associations were found between specific IgE and IgG1 or IgG4 antibodies (p <0.001, p =0.004). ELISA inhibition tests showed significant inhibitions by both free cefalcor and cefaclor-HSA conjugate.

## Conclusions

Most common manifestation of immediate hypersensitivities to cefaclor was anaphylaxis in which IgE mediate response is the major pathogenic mechanism. Detection of serum specific E and IgG subtypes to cefaclor-HSA conjugate may be useful to diagnose cefaclor anaphylaxis. The IgG subtype- mediated response was suggested in some cases of cefaclor anaphylaxis.

